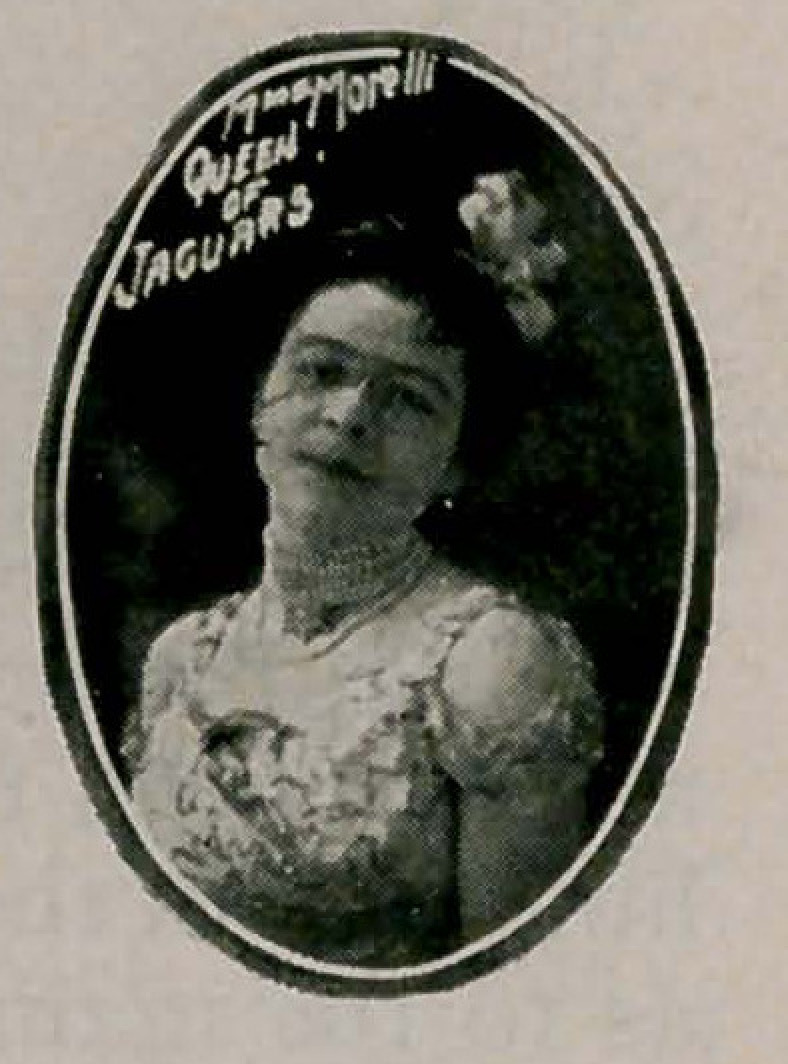# Pan-American Notes

**Published:** 1901-10

**Authors:** 


### PAN-AMERICAN NOTES.

MR. FRANK C. BOSTOCK'S animal arena is one of the most attractive exhibitions of the animal kingdom that the world has ever witnessed. It is not only entertaining, but it is instructive, and has already been the means of informing hundreds of thousands of young people as to the manner of training wild and ferocious animals, and also as to their habitats, methods of life, and general conduct towards human beings. Especially has it taught the young mind the necessity of kindness in dealing with all animal nature.

THE GREAT BOSTOCK.

A sketch of Mr. Bostock's career may prove of interest. He was born in a traveling caravan in England and is now 35 years of age. He began to train animals at the age of 14, a mere lad, and has attained the front rank. He has justly won the name of "The Animal King," to which his long experience and success as a trainer entitle him. He is in the fresh vigor of young manhood, and is one of the most accomplished showmen in the world. His Pan-American Exposition arena alone cost half a million of dollars, and altogether his enterprises, here and elsewhere, aggregate a cost of a million or two of money.

BONAVITA'S MASTERPIECE.

He lost, by a great fire at Baltimore last winter, his eastern "zoo," in which there were 52 lions, and a full assortment of animals gathered from all parts of the world. His agents in various countries soon replaced the animals and drawing upon his Indianapolis and Milwaukee menageries, he was able to establish at the exposition on May 1, the greatest animal exhibition in the world.

Captain Jack Bonavita, "the lion monarch," as he has been appropriately named, is one of the greatest trainers of lions, and has taken as many as 27 of these ferocious beasts into the arena at one time. Occasionally he has prolonged contests with some of them before he succeeds in making them take their proper places, and sometimes receives wounds while performing his great lion acts. He is 36 years old and converses most interestingly and agreeably on the subject of his life-work.

Madame Morelli is another wonderful instance of human mastery over the most ferocious of wild beasts. She and her leopards, jaguars and panthers make a thrilling exhibition that is as fascinating as it is novel. The whole management is characterised by the utmost discipline and every employee in Mr. Bostock's great zoological garden appears to have acquired by the example of the chief, those courteous manners that are so essential to success. No visitors at the exposition will have done justice to themselves without seeing this greatest of menageries.

## Figures and Tables

**Figure f1:**
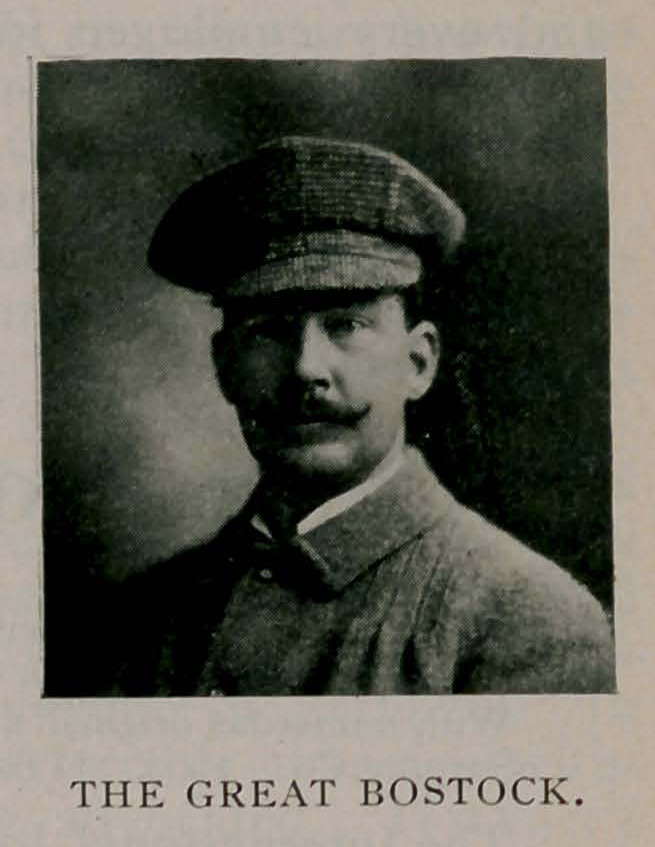


**Figure f2:**
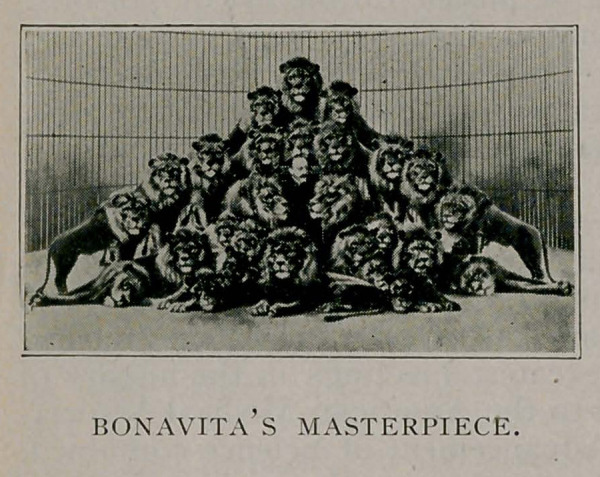


**Figure f3:**